# Assessment of mask-wearing adherence and social distancing compliance in public passengers in Hamadan, Iran, during the COVID-19 pandemic

**DOI:** 10.34172/jrhs.2021.61

**Published:** 2021-08-30

**Authors:** Ali Reza Soltanian, Tahereh Omidi, Salman Khazaei, Saeid Bashirian, Rashid Heidarimoghadam, Ensiyeh Jenabi, Maryam Mohammadian-khoshnoud

**Affiliations:** ^1^Modeling of Noncommunicable Diseases Research Center, Hamadan University of Medical Sciences, Hamadan, Iran; ^2^Student Research Committee, Hamadan University of Medical Sciences, Hamadan, Iran; ^3^Research Center for Health Sciences, Hamadan University of Medical Sciences, Hamadan, Iran; ^4^Social Determinants of Health Research Center, Hamadan University of medical sciences, Hamadan, Iran; ^5^Department of Ergonomy, School of Public Health, Hamadan University of Medical Sciences, Hamadan, Iran; ^6^Autism Spectrum Disorders Research Center, Hamadan University of Medical Sciences, Hamadan, Iran; ^7^Department of Biostatistics, School of Public Health, Hamadan University of Medical Sciences, Hamadan, Iran

**Keywords:** SARS-CoV-2, Masks, Social distancing, Treatment adherence and compliance

## Abstract

**Background:** The determination of the rate of social distancing compliance and mask-wearing adherence is essential to address the health aspects of COVID-19. The present study aimed to estimate the adherence to mask-wearing and maintaining the social distancing in public passengers in Hamadan, west of Iran, during the COVID-19 pandemic.

**Study design:** The present study was conducted based on a cross-sectional design.

**Methods:** The current study included 72 images from 12 areas in Hamadan as clusters in January 2021. The images were extracted from the traffic control center of Hamadan Municipality. The mean of social distancing and percentage of mask-wearing in all clusters were obtained based on cluster sampling.

**Results:** In this research, the majority of people(68%) in public passengers were men and 32% women. The mean±SD of social distancing in all public passengers in Hamadan was obtained at 65.27 ±73.37 cm (95% CI: 38.48-92.08 cm). The percentage of men who wore masks correctly was higher than that of women (57% vs. 51%). Moreover, mask-wearing adherence was not recognizable in about 34% of people in the images (28% of men versus 48% of women). Among the people whose images were recognizable, all the women were wearing masks, while about 13% of men were not (*P*<0.05).

**Conclusion:** As evidenced by the obtained results, the mean social distance in Hamadan was much lower than the standard value (1.5-2 meters) even at the time of restrictions. Although more than half of people wore masks in public passengers, it was much less than that in developed countries. Therefore, people should pay more attention to health advice regarding mask-wearing and maintaining social distance.

## Introduction

Since the coronavirus outbreak in January 2020 in Wuhan, China, drug therapies have been largely supportive, and vaccination has recently come for help in disease control. The COVID-19 pandemic has led to significant death tolls across the globe, posing unprecedented economic and social problems for almost all countries. Coronaviruses are spherical particles about 100 nm in diameter that can be easily transmitted via respiratory droplets due to their small size ^1^.

Evidence suggests that coronaviruses can be active and suspended in the air ^2^. The easiest way to prevent the transmission of airborne viruses is wearing masks and maintaining enough distance from each other. Recent research has revealed that cloth masks have poor filtration efficiency and are unlikely to control COVID -19 ^3,4^. Although indoor spaces without proper ventilation have a higher risk of person-to-person transmission, we should bear in mind that the risk of disease transmission will be also high in open spaces if the adequate social distance is not maintained. The World Health Organization (WHO) has issued outbreak regulations for countries and regions around the world. These laws are mostly in the form of restriction of movement and social interactions, short-term quarantine, adherence to social distancing, and use of face masks ^5,6^.

 The rate of adherence to proper mask-wearing and also adequate social distancing during the COVID-19 outbreak as effective factors in the spread of the disease has not been continuously measured and scheduled in different societies. Nonetheless, the knowledge and awareness of governments about public adherence to correct use of masks, as well as social distancing compliance in shopping centers, stations, and crowded places can be of great help in better planning for disease control.

Various mathematical models have been proposed by researchers to estimate the social distance ^7-9^, and some studies have even used mobile tracking to assess this distance ^7^. Such methods require advanced imaging facilities and rapid processing; therefore, they may not be feasible in developing or overcrowded countries due to their high cost. The present study strived to propose a simple method available in all countries to estimate face mask use and the mean social distance. One of the tools that can address these two issues is city-wide traffic control cameras. The current research used traffic control cameras to take high-quality photos from selected places in Hamadan; subsequently, the images were analyzed and processed using computer software programs. The study was conducted to estimate the rate of adherence to correct use of face masks and social distancing compliance in the public passengers of Hamadan, west of Iran.

## Methods

 This present cross-sectional study was approved by the Research Ethics Committee of Hamadan University of Medical Sciences (IR.UMSHA.REC.1399.683), Iran. The images were extracted from the traffic control center of Hamadan Municipality in coordination with the Hamadan University of Medical Sciences in January 2021. To assess the social distance and correct use of face masks, 12 areas in Hamadan which were covered by traffic control cameras were selected. These 12 regions were regarded as clusters. To take images, three days in January 2021 were randomly selected. Thereafter, 72 images were taken from these areas (i.e., 12^areas^× 3^days^×2^time(at 10 and 12 am)^).

###  Social distance assessment

**Figure 1:**
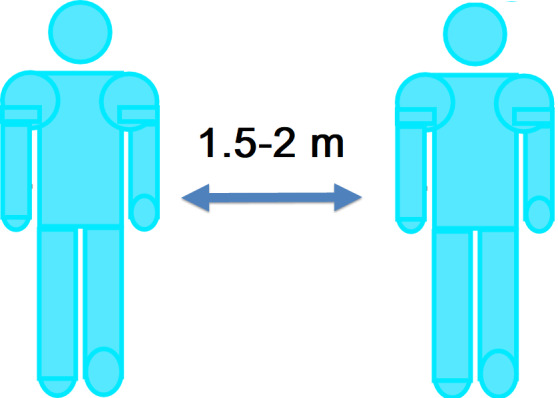
Standard social distance between two people as recommended by WHO

In the present study, ImageJ software (version 1.52) was used for the assessment of social distance. To calculate the distances in this software, the map scale and the magnification rate must be introduced. Therefore, before taking images, the scales were identified and introduced to the software using line drawing and marking of those areas. In each image, the distance between two people was calculated. As illustrated in Figure 1, the closest part of the body to each other was considered the social distance between individuals. The standard social distance is 1.5- 2 meters, and in the current study, the distance between people was measured in centimeters ^10^.

###  Evaluation of wearing masks 

**Figure 2:**
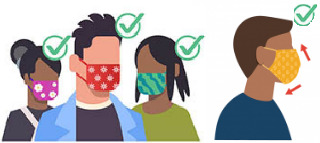
Correct way to wear a mask according to the CDC recommendation

To measure the use of masks by the general population, the number of gender-unspecified individuals with correct mask-wearing, incorrect mask-wearing, and without masks was recorded. The correct way to wear masks is depicted in Figure 2. If the person was wearing a mask but did not conform to Figure 2, it was regarded as the incorrect use of the mask. Some people also covered their faces with scarves due to the cold weather, and these cases were considered unrecognizable. Moreover, in some images, due to the angle of the person in the image, the face of the person was not visible and did not allow us to check the use of mask; therefore, these people were also registered as unspecified masks. After imaging and magnifying them, each image was evaluated and coded independently by two experts. The camera height, as well as the quality and resolution of all images, were the same.

###  Statistical analysis

 In the current study, the cluster sampling method was used so that each imaging area was considered a cluster. Since the number of people in the images was not equal, the clusters were considered to be of unequal sample size. The mean and standard deviation of social distance between individuals in each area were obtained using the following formula:


$${\bar{Y}}_{n}=\frac{1}{n}\sum_{i=1}^{n}\frac{M_{i}}{\bar{M}}{\bar{Y}}_{i}$$

where *Mi* is the number of people in each cluster,

$$\bar{M}=\frac{M}{N}$$

is the average of the number of people in all areas,

$$M=\sum_{i=1}^{N}m_{i}$$

is the number of elements in the population, N is the number of clusters in the population,N is the average of social distance in each cluster, and *n* is the number of selected areas. It should be noted that the average cluster size for the population (i.e., M) can be estimated

$$\bar{m}$$

if M is unknown. In the present study,

$$\bar{m}$$

is the average of all people in 12 clusters (areas).

 The variance of

$${\bar{Y}}_{i.}$$

is obtained using the following equation:


$$\mathrm{var}\left({\bar{Y}}_{n}\right)=\left(\frac{1}{n}-\frac{1}{N}\right)\left[\frac{1}{n-1}\sum_{i=1}^{n}{\left(\frac{M_{i}}{\bar{M}}{\bar{Y}}_{i}-{\bar{Y}}_{n}\right)}^{2}\right]$$

In addition, the following equations were used to determine the average percentage of mask-wearing in all clusters (areas).


$${\bar{P}}_{n}=\frac{1}{n}\sum_{i=1}^{n}\frac{M_{i}}{\bar{M}}P_{i}$$

$$\mathrm{var}\left({\bar{P}}_{n}\right)=\left(\frac{1}{n}-\frac{1}{N}\right)\left[\frac{1}{n-1}\sum_{i=1}^{n}{\left(\frac{M_{i}}{\bar{M}}P_{i}-{\bar{P}}_{n}\right)}^{2}\right]$$

where,

$$P_{i}$$

is the percentage of wearing a mask in the area i^th^, and the rest of the notations are mentioned above. The independent t-test was used to compare the mean of a quantitative variable in two independent populations, and Z-test was employed to compare the proportions in two independent populations. For image analysis, three packages of software, including Excel 2016, R (version 4.0.2), and ImageJ (version 1.52) were used.

## Results

 In the present study, 12 areas of Hamadan in the west of Iran were selected to assess adherence to mask-wearing and social distancing compliance in the public passengers of the city. To this end, a total of 72 images were evaluated. In this research, 68% of people in public passengers s in Hamadan were male, and 32% were female, and the presence of men was about 2 times as much as that of women during the restriction period. The mean of social distancing adherence in all public passengers in Hamadan (65.27±73.37 cm, 95% CI=38.48, 92.08 cm) was less than the standard value in January 2021 (150-200 cm). The mean social distancing compliance in all public passengers in Hamadan was less than 150 cm, and only in three places, it was between 100 and 140 cm.

 About half of people in Hamadan (55.12%; CI 95%=46.72-63.52%) were wearing masks correctly. Moreover, the percentage of men (57.23%; CI 95%=49.72-68.74%) who wore masks correctly was higher than women (51.01%; CI 95%= 39.61-62.41%); nonetheless, this difference was not statistically significant (P=0.47). Furthermore, the use of face masks was not visible in 34.3% (CI 95%=25.41-43.19%) of people (28% of men versus 48% of women). This can be ascribed to the cases when the head was bent, the person was backward, or there was an obstacle in front of the person's face (e.g., wearing scarves) during the shooting.

 In the present study, out of the people whose images were recognizable, there was no woman without a mask, while about 13% of men did not wear a mask (P<0.05). In general, the results of the present study pointed out that 8.26% (CI 95% = 2.7, 15.3%) of people did not wear masks in public passengers, and 2.31% of used masks incorrectly (CI 95%= 0.16-4.48%).

## Discussion

 The rapid spread of COVID-19 at the community level has necessitated the implementation of behavioral disease prevention measures. According to the published reports, the use of face masks and maintaining social distancing by the general public is effective in mitigating the prevalence of COVID-19. Public adherence to these preventive behaviors is critical to slow the spread of COVID-19 infection in the community. One of the most effective measures to slow the spread of COVID-19 is adherence to adequate social distancing and the correct use of face masks in public passengers. An awareness of the implementation of these two measures as reliable indicators can be used to decide on the imposition, extension, or removal of restrictions. It is hoped that the present study will introduce a simple method for the estimation of mask-wearing adherence and mean social distancing compliance in society to help health policymakers take appropriate measures for disease control.

###  Mask-wearing adherence

The present study demonstrated that about one year after the observation of the first COVID-19 patient in Iran, about half of pedestrians (55%) in the streets of Hamadan wore masks correctly, which is relatively less than that in some reports. For instance, in a report issued in eight US states, 75% of people were adherent to mask-wearing 12. The rate of mask-wearing in Hamadan (west of Iran) was very lower, as compared to that in Japan 13 and Vietnam 14(99.5%), as well as Hong Kong (96.4%) according to residents' self-reports. Nevertheless, the rate of correct mask-wearing in Hamadan was higher than that reported in the study by Haischer et al. ^15^ in Wisconsin (41%).

 This discrepancy can be attributed to the time of the study, the prevalence rate of the disease, and the cultural issues related to adherence to health practices, the economic status of households (families) in providing masks, government health policies, or even the normalization of the disease in society. The current study did not examine the relationship of mask-wearing adherence with economic, social, and cultural status, as well as other factors. Therefore, it is recommended that future research address the impact of such factors on mask-wearing adherence.

 Although no pedestrians without masks were observed in this study, it is noteworthy that a percentage of women were invisible due to head lowering, being behind the camera, and covering their faces with scarves. Therefore, it is not safe to arrive at the conclusion that the proportion of pedestrians without masks in public passengers in Hamadan is negligible. Furthermore, the obtained results indicated that approximately 2% of pedestrians wore the mask incorrectly, which is very low, suggesting that people are properly informed and educated about the correct use of face masks.

Furthermore, it was found that approximately one-tenth of the general population did not demonstrate mask-wearing behavior. Given the restrictions imposed in the winter, which is also the peak time of infectious-respiratory diseases, it seems to be a significant amount. This rate is higher, as compared to the findings of other studies, especially in East Asian countries ^13,14^.

###  Social distance adherence

In the present study, the mean social distancing compliance was less than the value recommended by the WHO. However, in a study conducted in Ireland ^8^, there was a notable adherence to social distancing; therefore, 83.7% of people observed two-meter social distancing rule and 55.9% of them avoided meetings. The results of previous studies indicated that factors, such as income, race, education, and also the availability of health services, justify the variations in social distancing among different communities ^16-18^.

In the current study, since the images were captured by cameras, it was not possible to investigate the factors related to social distancing compliance and mask-wearing adherence through interviews with citizens. An intervention study in Berlin, Germany, reported that distances of 161.7 and 152.7 cm need to be maintained from masked and unmasked people, respectively ^10^. As demonstrated, the mean social distancing compliance in Berlin is higher than that in Hamadan. This low level of social distancing in Hamadan can be ascribed to taking pictures from the main and busy public passengers of Hamadan.

 The identification of the determinants of social distancing adherence and mask-wearing is essential to address the health and economic aspects of COVID-19. Compliance with protective behaviors that mitigate the spread of COVID-19 can vary significantly from person to person in different communities. This discrepancy can be reflected in differences in perceived risk, the cost of social distancing, social norms, and even attitudes toward health policymakers' recommendations. Individual adherence to health guidelines can also be affected by the degree of confidence or trust in health policymakers. Therefore, it is suggested that in future studies, apart from estimating the rate of adherence to preventive behaviors, its determining factors also be examined.

 Among the notable limitations of the present study, we can refer to the impossibility of taking pictures in the afternoon due to short days in winter. Moreover, to provide high resolution and high-quality images, the images were forced to be captured in a clear situation. Furthermore, it was not feasible to interview people and determine the factors that affect the rate of people’s adherence to preventive behaviors.

## Conclusion

 As evidenced by the obtained results, the mean social distance in Hamadan was much lower than the standard value (1.5-2 meters) even at the time of restrictions. Although more than half of people wore masks in public passengers, it was much less than that in developed countries. Therefore, people should devote more attention to health advice regarding mask-wearing and social distancing compliance

## Acknowledgments

 The present study was funded by the Deputy of Research and Technology of Hamadan University of Medical Sciences, Hamadan, Iran. The deepest appreciation goes to Hamadan Traffic Control Center for assisting us in this research project.

## Conflict of interests

 The authors declare that they have no conflict of interest.

## Funding

 The study was funded by the Hamadan University of Medical Sciences, Hamadan, Iran (No. 9908205896).

## Ethics approval

 The study protocol was approved by the Ethics Committee of Hamadan University of Medical Sciences, Hamadan, Iran (IR.UMSHA.REC.1399.683).

## Highlights

- Social distancing compliance in Hamadan was lower than the standard value recommended by the world health organization.
- Mask-wearing adherence in public passengers in Hamadan was about 50%.
- The percentage rates of correct mask-wearing in men and women were obtained at 57% and 51%, respectively.
